# Use of Cytomegalovirus Immunoglobulin with Antiviral Therapy for Cytomegalovirus Infection in Transplant Recipients: A Tertiary Care Single-Center Experience

**DOI:** 10.3390/v18060599

**Published:** 2026-05-25

**Authors:** Reem M. Alameer, Bayan Alamro, Khulud Alanazi, Ali Alahmari, Ghadeer Almousa, Abdullah Almohaizeie, Hadeel Samarkandi, Reem S. Almaghrabi

**Affiliations:** 1 Section of Transplant Infectious Diseases, Organ Transplant Center of Excellence, King Faisal Specialist Hospital and Research Center, Makkah Al Mukarramah Branch Road, Al Maather, Riyadh 12713, Saudi Arabia; ramaghrabi@kfshrc.edu.sa; 2 Section of Infectious Diseases, Internal Medicine Department, King Faisal Specialist Hospital and Research Center, Riyadh 11211, Saudi Arabia; bayanalamr@gmail.com (B.A.); kalanazi2@kfshrc.edu.sa (K.A.); 3 Department of Hematology, Stem Cell Transplantation & Cellular Therapy, Cancer Centre of Excellence, King Faisal Specialist Hospital and Research Center, Riyadh 11211, Saudi Arabia; aalahmari7@kfshrc.edu.sa (A.A.); hsamarkandi61@kfshrc.edu.sa (H.S.); 4 Pharmaceutical Care Division, King Faisal Specialist Hospital and Research Center, Riyadh 12713, Saudi Arabia; galmousa@kfshrc.edu.sa (G.A.); amohaizeie@kfshrc.edu.sa (A.A.); 5 College of Pharmacy, Alfaisal University, Riyadh 11533, Saudi Arabia

**Keywords:** Cytomegalovirus, solid organ transplant, hematopoietic stem cell transplant, and chimeric antigen receptor T-cell (CAR-T) therapy, CMV immunoglobulin

## Abstract

Background Cytomegalovirus (CMV) infection is a major contributor to morbidity and mortality in recipients of hematopoietic stem cell transplant (HSCT), solid organ transplant (SOT), and chimeric antigen receptor T-cell (CAR-T) therapy. While antiviral agents remain the cornerstone of treatment, CMV-specific immunoglobulins (CMVIG) have been utilized as adjunct therapy with variable outcomes. This study aims to evaluate the virological response and tolerability of CMVIG in cases of severe or refractory CMV viremia, with or without CMV disease. Methods: We conducted a single-center retrospective case series of adult recipients of SOT, allogeneic HSCT, and/or CAR-T cell therapy who developed CMV viremia or disease and received at least one dose of CMVIG between May 2017 and May 2023 at our center. Virological improvement within 14 days of starting CMVIG and tolerability of CMVIG are the primary outcome of this study. Results: A total of 33 patients were included. Of these, 29 underwent transplantation [SOT: 48.2%, HSCT: 51.7%], and five underwent CAR-T cell therapy (one post-HSCT). High-risk CMV serostatus was present in 12%. CMV viremia was documented in 32 patients (97%), and tissue-invasive disease was present in 11 patients (33.3%). Virological response, was observed in 65.6% of the cohort. The median time to undetectable CMV viral load following CMVIG initiation was 28 days. CMVIG was well-tolerated. All-cause mortality at 90 days remained high (57%). Conclusion: In this case series, CMVIG demonstrated a virological response rate of 65.6% in patients with severe or refractory CMV infection. While CMVIG was well-tolerated with minimal adverse events, the high mortality rate despite virological response suggests that CMVIG may be insufficient for this critically ill population. Our findings should be interpreted as observational data from a small case series, and prospective controlled trials are needed to establish the true benefit of CMVIG in combination with standard antiviral therapy.

## 1. Introduction

Cytomegalovirus (CMV) infection is a well-known contributor to mortality and morbidity after hematopoietic stem cell transplant (HSCT), chimeric antigen receptor T-cell (CAR-T) therapy, and solid organ transplant (SOT) [1,2,3,4]. The incidence of CMV infection varies by transplant type and donor–recipient serostatus, ranging from 9% to 50% in HSCT recipients and 5% to 15% in SOT recipients [5,6]. In CAR-T cell therapy recipients, CMV reactivation occurs in approximately 15–27% of patients, particularly during the cytokine release syndrome phase and subsequent immune reconstitution period [7].

CMV has both direct effects of viremia and end-organ damage and indirect effects that include immunomodulatory properties leading to risk of graft loss, graft vs. host disease (GVHD), and increased risk of invasive bacterial and fungal infections [1,8,9]. Prevention of CMV viremia and infection is key in the management of SOT and HSCT patients, especially in the first 100 days after transplant [10,11,12]. No clear guidance for CMV prophylaxis in case of CAR-T cell therapy recipients [7].

Despite available preventive measures, effective antiviral therapy, and even in the absence of genetic mutation (resistance) and due to high levels of T cell dysfunction and depletion post-transplant, CMV infection can be persistent and refractory to therapy [13]. Chemaly et al. had defined refractory CMV infection as failure of CMV viremia or CMV disease-related symptoms to improve after 2 weeks of appropriate antiviral therapy [13]. Antiviral therapy remains the cornerstone of therapy in the event of CMV infection [11]. Recent approvals of newer antiviral agents, including maribavir and letermovir, have expanded treatment options for CMV infection. However, these agents are not universally available, are associated with significant cost, and may have limited efficacy in patients with high viral loads or specific resistance mutations. Additionally, conventional antiviral agents are often associated with significant toxicities, including myelosuppression (ganciclovir), nephrotoxicity (foscarnet, cidofovir), and electrolyte abnormalities, which may limit their use in critically ill transplant recipients.

Addition of CMV-specific immunoglobulins (CMVIGs) have been described in the literature with variable outcomes when used as part of initial or salvage therapy [14,15,16,17,18,19,20]. Cytotect^®^ (Biotest AG, Dreieich, Germany) or Cytogam^®^ (CSL Behring, King of Prussia, PA, USA) are the commercially available CMVIGs that are licensed to be used for CMV prophylaxis. They act by neutralizing CMV and modulation of innate and adaptive immunity response to the virus [21]. It has been shown that CMVIG, when used as part of CMV prophylaxis in SOT recipients, improved overall survival rate and reduced CMV disease [22,23]. CMVIG is well-tolerated in most studies, with a minimal side effect profile compared to the more toxic impact of antiviral medications and other CMV active agents (mTOR inhibitors and leflunomide) [24].

We conducted a single-center retrospective case series to describe the virological response, safety profile, and clinical outcomes in patients with severe or refractory CMV infection who received CMVIG as adjunctive therapy to standard antiviral treatment. We sought to characterize the patient population, treatment patterns, and outcomes across SOT, HSCT recipients and CAR-T therapy to provide evidence on CMVIG use in this high-risk population.

## 2. Methods

### 2.1. Study Design

We conducted a single-center retrospective case series of consecutive patients who received CMVIG as adjunctive therapy for severe or refractory CMV infection at King Faisal Specialist Hospital and Research Center (KFSH&RC),Riyadh, Saudi Arabia; a tertiary care center providing comprehensive transplantation services including SOT, HSCT and CAR-T cell therapy.

### 2.2. Data Collection

#### 2.2.1. Patient Population and Inclusion/Exclusion Criteria

Inclusion criteria were: (1) age ≥ 18 years, (2) confirmed CMV infection (viremia defined as detectable CMV DNA by quantitative PCR or end-organ disease documented by histology, culture, or clinical/radiological findings), (3) receipt of CMVIG as adjunctive therapy for treatment of CMV infection, and (4) available baseline and follow-up CMV viral load data for at least 14 days following CMVIG initiation.

Exclusion criteria were: (1) CMVIG administered for CMV prophylaxis only, (2) lack of documented CMV infection, (3) incomplete follow-up data, and (4) age < 18 years.

#### 2.2.2. CMVIG Administration Protocol

CMVIG was administered intravenously at a dose of 2–3 mL/kg (100–150 mg/kg) as Cytotect CP (Biotest, Dreieich, Germany). CMVIG was administered to patients with severe CMV infection (tissue-invasive disease) or refractory CMV infection (viremia or disease persisting despite ≥2 weeks of standard antiviral therapy). The decision to initiate CMVIG was made by the treating physician in consultation with the infectious diseases team. CMVIG was typically given in conjunction with ongoing standard antiviral therapy.

#### 2.2.3. Population Investigated/Case Definition (See Supplementary Materials)

Therapy definition: any patient who meets the inclusion criteria, who received one or more doses of CMVIG (Cytotect^®^) −/+ antiviral therapy. CMV case definition (Ljungman P et al. Clin Infect Dis 2017) [25]: see Supplementary Materials. CMV refractory or resistance (Chemaly et al. Clin Infect Dis 2019) [13]: see Supplementary Materials.

CMV viral load was quantified using the Abbott Alinity m CMV Assay (Abbott Molecular, Abbott Park, IL, USA), a real-time PCR assay with a quantitative range of 1.49 to 8.19 log_10_ IU/mL and a lower limit of quantification of 1.48 log_10_ IU/mL. Results were re-ported in log_10_ IU/mL, and virological response was defined as a ≥1 log_10_ reduction in CMV DNA from baseline to day 14 [26]. (see Supplementary Materials).

#### 2.2.4. CMV Prevention and Treatment Protocols

CMV management protocols remained consistent throughout the study period (2017–2022). All SOT recipients who were CMV D+/R− or R+ received universal CMV prophylaxis with valganciclovir for 3–6 months post-transplant. HSCT recipients received CMV prophylaxis or pre-emptive therapy based on institutional protocol and donor–recipient serostatus. First-line treatment for CMV viremia was intravenous ganciclovir at 5 mg/kg twice daily. Alternative agents (valganciclovir, foscarnet, cidofovir) were used for patients with ganciclovir resistance, intolerance, or renal dysfunction. There was no CMV prophylaxis for CAR-T recipients in our institution; however, we used pre-emptive therapy based on viral load monitoring and clinical risk factors.

#### 2.2.5. Outcomes and Definitions

The primary outcome was virological response, defined as ≥1 log (10-fold) reduction in CMV viral load within 14 days of CMVIG initiation. Secondary outcomes included: (1) time to complete virological response (negative CMV PCR), (2) clinical response (resolution of CMV-related symptoms or improvement in organ function), (3) adverse events related to CMVIG, and (4) all-cause mortality at 90 days post-CMVIG initiation.

### 2.3. Statistical Analysis

Continuous variables were summarized as median or interquartile range and will be compared using the *t*-test or Mann–Whitney test. Categorical variables are presented as numbers and percentages (n, %). As this is a retrospective chart review, consent was waived.

## 3. Results

From 2017 to 2023, a total of 33 patients received CMVIG as part of CMV therapy. Of these, 29 underwent transplantation [SOT: 48.2%, HSCT: 51.7%], and five underwent CAR-T cell therapy (one post-HSCT). Overall, 45.4% were female, and the median age was 35.9 years (IQR 24–53 years). High-risk CMV serostatus—donor-positive/recipient-negative (D+/R−) for SOT or donor-negative/recipient-positive (D−/R+) for HSCT—was present in 12% of the total population (Table 1).

CMV viremia was documented in 31 patients (94%), and tissue-invasive disease was present in 11 patients (33.3%). All patients received antiviral therapy alongside CMVIG. Virological response was observed in 65.6% of the cohort. The median time to undetectable CMV viral load following CMVIG initiation was 28 days. CMVIG was well-tolerated, with no infusion-related adverse events reported. All-cause mortality at 90 days 19 out of 33 (57%) (Table 2).

### 3.1. Solid Organ Transplant (SOT) Recipients (Supplementary Table S1)

A total of 14 SOT recipients were included in the analysis: six small bowel, four kidney, three liver, and one bilateral lung transplant. The majority of patients (9 out of 14) received T cell-depleting agents (either alemtuzumab or anti-thymocyte globulin [ATG]) as induction therapy at the time of transplant. For maintenance immunosuppression, recipients were managed with a regimen consisting of two to three agents, typically including a calcineurin inhibitor (tacrolimus, FK), an antimetabolite (mycophenolate mofetil, MMF), and corticosteroids. Only 3 of the 14 recipients were maintained on an mTOR inhibitor. CMV serostatus mismatch (donor-positive/recipient-negative [D+/R−]) was observed in three recipients (two small bowel and one kidney), while the remaining 11 out of 14 patients were CMV-seropositive (R+).

CMV viremia was documented in 13 of the 14 solid organ transplant (SOT) recipients at the time of CMV infection diagnosis. The median peak CMV viral load among these 13 patients was approximately 15,354 IU/mL. Tissue-invasive disease was identified in 8 out of 14 recipients based on histopathology, with the majority presenting with gastrointestinal involvement—manifesting as colitis, enteritis, or esophagitis. Additionally, one patient had CMV hepatitis, two patients developed CMV pneumonitis, and two patients had multi-organ involvement. Most patients presented with nonspecific symptoms at the time of tissue-invasive disease diagnosis, including fever, fatigue, and gastrointestinal symptoms. The mean time from transplant to CMV diagnosis among the 14 patients was approximately 269 days, and the median time was 167 days (IQR 1–1180). There was no augmentation of immunosuppression around the time of CMV diagnosis, with the exception of one patient who received infliximab before diagnosis with CMV disease by 2 weeks (#21). Interestingly, 30 days prior to CMV diagnosis, 3 out of 14 SOT recipients had an absolute lymphocyte count (ALC) below 1000 × 10^9^/L. However, at time of diagnosis, 7 out of 14 had a very low ALC of less than 500 × 10^9^/L.

All 14 patients received at least one dose of CMVIG as part of their CMV infection treatment, administered in combination with antiviral agents (valganciclovir and ganciclovir). CMVIG was indicated in cases of severe CMV infection with tissue-invasive disease or in refractory infections. CMV genotyping was performed in 3 of the 14 patients (#21, #33 and #34), with resistance mutations identified in two (#33 and #34) (UL54 [A692 gene] and UL97). Both patients with resistance were small bowel transplant recipients. One was switched from ganciclovir to foscarnet, while the other received combination therapy with ganciclovir and foscarnet. Notably, both cases were managed prior to the approval of maribavir in 2021. One patient who was diagnosed in 2023 with severe refractory CMV infection with no detected resistance was managed with a combination of foscarnet and marabivir, along with CMVIG (#21).

Virological response among SOT recipients, defined as ≥1 log reduction in viral load within 14 days, was observed in 8 out of 14 (57.14%) of the cohort. The rest had unchanged viral load and only one patient had further worsening of the viral load at day 14 of therapy (#15). Among SOT recipients with available data, the median time from CMV infection to undetectable viral load was 61 days (IQR 24.5–121.5). Four patients reported symptoms improvement while on CMVIG. CMVIG was generally well-tolerated, with only 1 out of 14 patients experiencing a mild adverse event characterized by chills and rigors that did not necessitate discontinuation of CMVIG.

All-cause mortality at 90 days following CMV diagnosis among SOT recipients occurred in 10 out of 14 patients (71.4%). Biopsy-proven acute graft rejection occurred in 8 out of 14 patients (57.14%) after the diagnosis of CMV infection (four small bowel, two kidney, one lung, one liver). Management of rejection included escalation of immunosuppression with therapies such as corticosteroids, intravenous immunoglobulin (IVIG), plasmapheresis, and ATG. Secondary infectious complications were documented in 11 out of 14 patients (78.56%), including bacterial and invasive fungal infections. Bacterial pathogens included Escherichia coli (n = 3), Klebsiella pneumoniae (n = 1), Morganella morganii (n = 1), Pseudomonas aeruginosa (n = 1), and Enterococcus faecium (n = 1), presenting as bacteremia, pneumonia, or urinary tract infection. Fungal infections included invasive candidiasis (n = 3) and probable Aspergillus infection (n = 1). One patient had polymicrobial infection with concurrent Escherichia coli bacteremia and invasive candidiasis. None of the patients included in this cohort diagnosed with post-transplant lymphoproliferative disorder (PTLD).

### 3.2. Hematopoietic Stem Cell Transplant (HSCT) (Supplementary Table S2)

A total of 15 HSCT recipients were included in the analysis: eight underwent matched related donor allogeneic HSCT, six received haploidentical transplants, and one received a matched unrelated donor (MUD) transplant. The most common underlying condition leading to transplantation was acute myeloid leukemia (AML). One patient received CAR-T cell therapy before HSCT (#12). Regarding conditioning regimens, all patients received a myeloablative regimen; however, four patients received reduced-intensity chemotherapy (RIC). For GVHD prophylaxis, recipients were placed on a regimen consisting of two agents, typically including FK, MMF, sirolimus, and others. CMV serostatus mismatch (D−/R+) was observed in one recipient, while the remaining patients, 14 out of 15, were donor CMV-seropositive (D+).

CMV viremia was documented in all 15 patients at the time of CMV infection diagnosis. The median peak CMV viral load among these 15 patients was approximately 4231 IU/mL. Only three patients were diagnosed with tissue-invasive disease (one encephalitis, one pneumonitis and one colitis/pneumonitis). Most patients had no to mild symptoms at time of CMV infection diagnosis. The mean time from transplant to CMV diagnosis was approximately 93.5 days (IQR 13 to 315). CMV infection occurred before engraftment in five patients (#12 #17 #18 #20 #27).

All 15 patients received at least one dose of CMVIG as part of their CMV infection treatment, administered in combination with antiviral agents (majority ganciclovir and foscarnet). CMVIG was indicated in cases of severe CMV infection with tissue-invasive disease or in refractory infections. CMV genotyping was performed in only one patient, which resulted in no resistant mutation identified (#26).

Virological response among HSCT recipients, defined as ≥1 log reduction in viral load within 14 days, was observed in 9 out of 15 patients (60%), with three patients reaching undetectable viral load at day 14 (#11 #14 #18). The rest had unchanged viral load, with none having a further increase in viral load. Among HSCT recipients with available data, the median time from CMV infection to undetectable viral load was 19 days (IQR 14.5–32). CMVIG was generally well-tolerated, with none of the 15 patients experiencing any adverse event.

All-cause mortality at 90 days following CMV diagnosis among HSCT recipients occurred in 6 out of 15 patients (40%). GVHD occurred in 11 out of 15 patients (73.33%) after the diagnosis of CMV infection (four biopsy-proven GI GVHD). It was largely managed with steroids and other immunosuppressive medications. Secondary infectious complications were documented in 10 out of 15 patients (66.66%), including bacterial and invasive fungal infections. Most patients had both bacterial and fungal infection at the same time. Bacterial pathogens included Klebsiella pneumoniae (n = 2), Enterococcus faecium (n = 1), and Enterobacter cloacae (n = 1), causing bacteremia and urinary tract infection. Fungal infections predominated, with probable invasive pulmonary aspergillosis (n = 5) and invasive candidiasis (n = 2). Additional complications included pulmonary Pneumocystis jirovecii pneumonia infection. One patient developed tonsillar PTLD after CMV diagnosis (#8).

### 3.3. Chimeric Antigen Receptor T-Cell (CAR-T) Therapy (Supplementary Table S3)

A total of four CAR-T therapy patients were included in the analysis. One patient underwent HSCT following CAR-T cell therapy (D+/R+) (#12) and is included in the HSCT analysis as CMV infection occurred after HSCT. All patients received fludarabine and cyclophosphamide (Flu/Cy) conditioning before cell infusion. The most common underlying condition leading to CAR-T cell therapy was diffuse large B cell lymphoma (DLBCL).

CMV viremia was documented in all four patients at the time of CMV infection diagnosis. The median peak of CMV viral load was 15,719 IU/mL. Three out of four patients had CMV viremia prior to CAR-T cell infusion (#6 (7 days) #23 (3 days) #24 (71 days)). None of the patients had tissue-invasive disease. At the time of CMV infection diagnosis, all four patients had an ALC of less than 500 × 10^9^/L.

All four patients received at least one dose of CMVIG as part of their CMV infection treatment, administered in combination with antiviral agents (majority ganciclovir and foscarnet). CMVIG was indicated in cases of severe refractory CMV infections.

Virological response among HSCT recipients, defined as ≥1 log reduction in viral load within 14 days, was observed in all four patients, with one patient reaching undetectable viral load at day 14 (#24). Among CAR-T cell therapy recipients, the median time from CMV infection to undetectable viral load was 83.5 days (IQR 33–119). CMVIG was generally well-tolerated, with no reported side effects.

All-cause mortality at 90 days following CMV diagnosis among CAR-T cell therapy patients occurred in three out of four patients. Secondary infectious complications were documented in three out of four patients, including bacterial and invasive fungal infections.

## 4. Discussion

This single-center retrospective case series describes our institutional experience with CMVIG as adjunctive therapy for severe or refractory CMV infection in a cohort of 33 patients including SOT, HSCT, and CAR-T recipients. Our principal finding is that the addition of CMVIG to antiviral therapy resulted in a virological response, defined as a ≥1 log reduction in viral load within 14 days, in 65.6% of patients. This finding is consistent with the existing literature and supports the continued use of CMVIG in complex clinical scenarios [18,27].

The patient population in our study represents a significant challenge in clinical practice, characterized by high-risk transplant types, profound immunosuppression, and multi-organ involvement. The use of CMVIG in our institution was reserved for these difficult-to-treat cases, including patients with refractory viremia, tissue-invasive disease, or intolerance to first-line antiviral agents. This approach aligns with the findings of Charlotte, R et al., who reported that 82% of CMVIG use in lung transplant recipients was for complex clinical situations arising from antiviral drug side effects [27]. Our study, therefore, provides valuable real-world evidence on the utility of CMVIG as a salvage therapy.

The virological response rate of 65.6% in our cohort is comparable to that reported in other studies of CMVIG for refractory CMV infection. For instance, Alsuliman et al. reported a 78% overall response rate in a multicenter study of 23 HSCT recipients receiving CMVIG as salvage therapy [18]. Although our response rate is slightly lower, this may be attributable to differences in patient populations, including the inclusion of SOT, HSCT and CAR-T recipients in our study, who may have different underlying risk factors and immune reconstitution kinetics. Nevertheless, our findings confirm that a substantial proportion of patients with refractory CMV infection can achieve virological control with the addition of CMVIG.

An important finding of our study is the excellent safety profile of CMVIG. Only one patient (3%) experienced a mild, transient adverse event (chills and rigors) that did not necessitate discontinuation of therapy. This is consistent with the findings of Alsuliman et al. and Charlotte et al., who also reported minimal adverse events [18,27]. The favorable safety profile of CMVIG is a significant advantage over conventional antiviral agents like ganciclovir and foscarnet, which are associated with significant toxicities such as myelosuppression and nephrotoxicity. In our cohort, CMVIG provided a therapeutic alternative for patients who had developed or were at high risk for antiviral-related toxicities.

Despite the encouraging virological response, the 90-day all-cause mortality in our cohort remained high at 57%. This sobering statistic likely reflects the severity of the underlying conditions and the profound immunosuppression in this patient population, rather than a failure of CMVIG therapy itself. Patients with refractory CMV infection are often critically ill with multiple comorbidities, and CMV infection is often a marker of poor overall immune function. Alsuliman et al. also reported a high 100-day mortality rate of 30.4% in their cohort of HSCT recipients with refractory CMV, despite a high virological response rate [18]. This underscores the fact that while CMVIG can help control CMV viremia, it may not be sufficient to reverse the cascade of complications in these high-risk patients.

The high mortality rate was particularly pronounced in our SOT subgroup (71.4%), which also had a high rate of acute graft rejection (57.14%). This highlights the complex interplay between CMV infection, immunosuppression, and graft function. CMV is known to have immunomodulatory effects that can increase the risk of graft rejection, while the augmented immunosuppression required to treat rejection can in turn exacerbate CMV infection [9,28]. The use of CMVIG, with its dual antiviral and immunomodulatory properties, may be particularly beneficial in this setting by helping to break this vicious cycle [29]. However earlier initiation of CMVIG might be the key to a better outcome.

An important observation in our SOT cohort was the progressive decline in absolute lymphocyte count (ALC) preceding and at the time of CMV diagnosis. Specifically, 30 days prior to CMV diagnosis, only 3 of 14 SOT recipients had an ALC below 1000 × 10^9^/L; however, at the time of CMV diagnosis, this number increased to 7 of 14 patients with an ALC of below 500 × 10^9^/L. This marked decline in lymphocyte count likely reflects severe T-cell depletion and is consistent with the known risk factors for CMV disease in transplant recipients. The profound lymphopenia observed in our cohort underscores the extreme immunosuppression present in these patients and may explain both the development of CMV disease and the high mortality rates despite virological response. The rapid progression to severe lymphopenia may also indicate that CMVIG, with its immunomodulatory properties, could be particularly valuable in this setting by providing immediate antiviral activity while immune reconstitution occurs. Future studies should investigate whether baseline or serial ALC measurements could serve as predictive markers for response to CMVIG therapy.

The role of CMVIG in the current era of newer antiviral agents, such as maribavir and letermovir, is an important consideration [30,31]. While these agents have expanded our therapeutic armamentarium, they are not without limitations, including cost, availability, and the potential for resistance. Maribavir is approved for refractory CMV, but its efficacy may be limited in patients with high viral loads or specific resistance mutations. Letermovir is primarily used for prophylaxis and is not approved for treatment. Therefore, CMVIG retains a valuable niche in the management of CMV infection, particularly for patients with antiviral resistance, intolerance, or in combination with other agents for a multi-targeted approach. Santhanakrishnan et al. have demonstrated the successful use of CMVIG in combination with leflunomide for the treatment of ganciclovir-resistant CMV in cardiothoracic transplant recipients, highlighting the potential for combination therapies [32].

Our study has several limitations that must be acknowledged. First, its retrospective, single-center design is subject to inherent biases, including selection bias and incomplete data collection. The small sample size, particularly in the CAR-T cell therapy subgroup (n = 4), limits the statistical power for subgroup analyses and the generalizability of our findings. The lack of a control group of patients who received antiviral therapy alone prevents us from definitively concluding that CMVIG provides an added benefit. However, given that all patients in our cohort had refractory or severe disease, it is ethically challenging to withhold a potentially beneficial therapy in a prospective trial. Finally, the heterogeneity of our patient population, including different transplant types and conditioning regimens, may have influenced the outcomes.

In conclusion, in this single-center retrospective case series, CMVIG demonstrated a virological response rate of 65.6% in patients with severe or refractory CMV infection. CMVIG was well-tolerated with minimal adverse events, making it a potentially useful adjunctive therapy for patients who are intolerant of or resistant to conventional antiviral agents. However, the high mortality rate (57% at 90 days) despite virological response suggests that CMVIG alone may be insufficient for this critically ill population. Our findings should be interpreted as observational data from a small case series rather than evidence of efficacy, as the lack of a control group prevents causal inference.

Future prospective, multicenter studies are needed to better define the optimal timing, dosing, and patient selection for CMVIG therapy in the context of newer antiviral agents. Additionally, studies investigating the role of immune reconstitution markers and CMV-specific T-cell responses in predicting CMVIG response would be valuable. Nevertheless, our findings support the continued investigation of CMVIG as a potential adjunctive tool in the management of severe or refractory CMV infection in transplant and CAR-T cell therapy recipients.

## Figures and Tables

**Table 1:** Basic characteristics.

Parameter	Total 33	
Basic characteristics		
Sex (M/F) n (%)	18 (54.5)/15 (45.5)	
Age Median (Range)	Median 35.9 years (IQR 24.4–53.0)	
CMV serology		
R+ n (%)	29 (87.8)	
* High risk (D+/R− OR D−/R+) n (%)	4 (12)	
Transplanted patients n (%)	29 (87.87)	
SOT recipients	14/29 (48.2%) Bilateral lung: 1 (Non CF—Bronchiectasis) Kidney: 4 (ESRD) Liver: 3 (1 Hepatitis B, 2 autoimmune hepatitis) Small bowel: 6 (short bowel syndrome)	
HSCT recipients	15/29 (51.7%) ^+^ Matched related donor allogeneic: 8 (3 SCD, 2 HL, 1 DLBCL, 1 CML and 1 MPAL) Haploidentical: 6 (2 ALL, 2 AML, 1 SCD and 1 AA) Matched unrelated: 1 (AML)	
Non-transplanted patients n (%)	4 (15)	
CAR-T cell therapy alone n (%)	4 (15) 3 DLBCL 1 High-grade B cell lymphoma progressed from FL	
Immunosuppression (IS)		
SOT recipient	Induction IS 4 Alemtuzumab + steroids 4 ATG + Steroids 4 Steroids + basiliximab 1 No induction 1 missing info	Maintenance IS 7 Prednisone + FK + MMF 3 Prednisone + FK 3 Prednisone + FK + Sirolimus 1 Prednisone
HSCT recipients	Conditioning: 11 Myeloablative 4 RIC	GVHD PPX 15 on at least two or more agents (FK, MMF, Methotrexate Cyclophosphamide, Cyclosporine and Sirolimus)
CAR-T cell therapy	5 received Flu/Cy	
CMV PPX at time of CMV diagnosis n (%)	19 (57) No CMV PPX 13 (39) GCV/VGC 1 (0.03) Letermovir	

R+: recipient CMV IgG-positive serology, D+/R−: Donor-positive CMV IgG and recipient-negative, ESRD: End stage renal disease, AA: Aplastic Anemia, MPAL: Mixed phenotypic Acute Leukemia, ALL: Acute lymphoid leukemia, AML: Acute myeloid leukemia, SCD: Sickle cell disease, HL: Hodgkins lymphoma, DLBCL: Diffuse large B cell lymphoma, CML: Chronic myeloid leukemia, SOT: solid organ transplant, HSCT: hematopoietic stem cell transplant, FL: Follicular lymphoma, Flu/Cy: fludarabine/cyclophosphamide, FK: Tacrolimus, MMF: Mycophenolate mofetil, PPX: prophylaxis, GCV: Ganciclovir, VGC: Valganciclovir. * High-risk serology patterns are defined by standard transplant practices: for HSCT recipients, donor CMV IgG non-reactive and recipient-reactive (D−/R+); for SOT recipients, donor-reactive and recipient non-reactive (D+/R−). ^+^ One patient underwent allogenic HSCT following CAR-T cell therapy (D+/R+) (#12) and is included in HSCT analysis as CMV infection occurred after HSCT and was not related to CAR-T therapy.

**Table 2:** Outcomes.

Parameter	Total 33
* CMV viremia n (%)	31 (93.9)
CMV viral load at diagnosis; median (IQR) IU/mL	2013 (1025–14,815.5)
Peak CMV viral load (median, IQR) IU/mL	9324 (2146–48,845)
CMV tissue-invasive disease n (%)	11 (33.3)
Organ/system involved n	9 GI Tract 5 Colon 4 Small bowel 1 Esophagus 4 Lung 1 CNS
More than one organ/system involved n (%)	3/11 (27) 1 Pneumonitis + Colitis (Haplo HSCT) 1 Pneumonitis + Colitis + Hepatitis (kidney) 1 Pneumonitis + Esophagitis (Bilateral lung)
Timing of CMV diagnosis	
Time between TX and CMV infection in days; median (range)	60.5 days (7–1179)
Time between TX and first dose CMVIG in days; median (range)	47.5 days (16–1192)
CMV therapy	
CMVIG used in combination with antiviral	33 (100)
Number of CMVIG doses; mean (range)	5 (1–39)
Antiviral therapy in combination with CMVIG; n (%)	18 (54) GCV monotherapy 11 (33) GCV + Foscarnet 4 (12) Other combination
CMV genotype testing; n (%)	4 (12)
CMV resistance detected	1 UL54 (A692) 1 UL97
Response to CMVIG	
At least one log reduction within 14 days of starting CMV IgG n (%)	21/32 (65.6)
Peak CMV VL for patients who responded within 14 days; median (IQR)	31,930 (87,939)
CMV VL at day 14 for patients who responded; median (IQR)	467.5 (1558.25)
Number of days until undetectable viral load from first CMVIG; median (range)	28 (17–73)
Outcome	
CMVIG side effects	1 Chills and rigors 0 Discontinuation
Secondary infection within 90 days of CMV diagnosis n (%)	24 (72.72%)
Graft rejection within 90 days of CMV diagnosis n (%)	13 (39)
All-cause mortality at 90 days of CMV diagnosis n (%)	19/33 (57) HSCT 6/15 SOT 10/14 CAR-T 4/5

* CMV viral load below 200 IU/ML is excluded.

## Data Availability

The original contributions presented in this study are included in the article/Supplementary Material. Further inquiries can be directed to the corresponding author.

## Supplementary Materials

The following supporting information can be downloaded at https://www.mdpi.com/article/10.3390/v18060599/s1, Table S1: SOT recipients’ individual characteristics and outcome; Table S2: HSCT recipients individual characteristics and outcome; Table S3: CAR-T cell therapy patients characteristics and outcome.
