# Supplementary material for: Use of Cytomegalovirus Immunoglobulin with Antiviral Therapy for Cytomegalovirus Infection in Transplant Recipients: A Tertiary Care Single-Center Experience
Source: Viruses. 2026 May 25;18(6):599. doi: 10.3390/v18060599 (PMC13307840; doi:10.3390/v18060599)
Supplement: Supplementary file 1 [file viruses-18-00599-s001.zip › viruses-4252460-supplementary.pdf]

Supplementary

Methods

Population Investigated/Case definition

Therapy definition: any patient who meets the inclusion criteria above, who received one or more doses of CMVIG (Cytotect® or Cytogam®) -/+ antiviral therapy.

CMV case definition (Ljungman P et al. Clin Infect Dis 2017) [25] (see supplementary)

- CMV infection is the detection of CMV nucleic acid in body fluid or tissue by using CMV PCR (more than 200 IU/Ml)
- CMV syndrome is the detection of CMV nucleic acid and 2 of the following
  - Fever, fatigability, atypical lymphocytes, neutropenia/leukopenia, thrombocytopenia and elevation of hepatic aminotransferases
- CMV disease/Tissue invasive: organ specific signs and symptoms along with detection of CMV from biopsy by rapid viral culture, immunhistochemistry, or DNA hybridization
- Histopathological evidence of CMV infection, characterized by cytopathic changes including enlarged cells (cytomegaly) and intranuclear and/or intracytoplasmic inclusions consistent with CMV. Diagnosis was confirmed by histopathology (H&E staining), immunohistochemistry (IHC) using anti-CMV antibodies, or in situ hybridization (ISH) when available

CMV refractory or resistance (Chemaly et al. Clin Infect Dis 2019)[13]

- Refractory CMV infection: CMV viremia that increases after at least 2 weeks of appropriately dosed antiviral therapy
- Probable refractory CMV infection: Persistent viral load after at least 2 weeks of appropriately dosed antiviral therapy
- Refractory CMV end-organ disease: Worsening in signs and symptoms or progression into end-organ disease after at least 2 weeks of appropriately dosed antiviral therapy
- Probable refractory CMV end-organ disease: Lack of improvement in signs and symptoms after at least 2 weeks of appropriately dosed antiviral drugs
- Antiviral drug resistance: Viral genetic alteration that decreases susceptibility to one or more antiviral drugs

Abbot Alinity M: CMV viral load was quantified using the Abbott Alinity m CMV Assay (Abbott Molecular, Abbott Park, IL, USA), a fully automated real-time PCR assay for the detection and quantification of cytomegalovirus (CMV) DNA in human plasma. The assay utilizes dual-target PCR technology (targeting UL34 and UL80.5 genes) and is standardized to the WHO International Standard for CMV. The quantitative range is 1.49 to 8.19 $\log_{10}$ IU/mL (approximately 31 to 155,000,000 IU/mL), with a lower limit of quantification (LLOQ) of 1.48 $\log_{10}$ IU/mL (approximately 30 IU/mL). All samples were

Table S1SOT recipients' individual characteristics and outcome

Patient #	Sex/age	CMV serostatus	Organ transplanted	T-cell induction	Days transplant to CMV infection	CMV viremia(Peak IU/ML) /Disease	Antiviral used	Days from CMV Diagnosis to CMVIG	Number of doses/Response at day 14	Time to undetected CMV	Graft rejection	Secondary infection	90 days mortality
#3	F/39	R+	Small intestine	Alemtuzumab	117	Yes(2,572)/	Ganciclovir	13	2/Unchanged	NA	Yes	Bacterial infection	Dead
#5	M/65	R+	Liver	-	78	Yes(257)/colitis	Ganciclovir Foscarnet Cidofovir	2	5/Undetected	14	No	IFI	Dead
#7	F/24	R+	Small intestine	Alemtuzumab	26	Yes(1,231)/	Ganciclovir Foscarnet	7	1/Unchanged	22	Yes	Bacterial infection	Alive
#10	F/43	R+	Liver	-	11	Yes(12,657)/	Ganciclovir	10	4/ >1 log reduction	NA	No	Viral infection	Dead
#15	M/73	R+	Kidney	-	35	Yes(2,013)/Esophagitis	Ganciclovir	1	4/>1 log increase	27	No	Bacterial infection	Dead
#19	F/21	D+/R-	Kidney	ATG	296	Yes(45,28235)/Hepatitis, enteritis, colitis & pneumonitis	Ganciclovir	1	5/>1 log reduction	101	No	Bacterial infection	Alive
#21	M/18	D+/R-	Small intestine	ATG	*0	Yes(234)/Enteritis	Ganciclovir Foscarnet Mirabavir	38	16/unchanged	142	Yes	Bacterial infection	Dead
#25	M/69	R+	Kidney	ATG	37	Yes(15,8366)/	Ganciclovir	12	4/>1 log reduction	45	Yes	Bacterial infection	Dead
#29	F/23	R+	Kidney	ATG	592	Yes(5,926)/	Ganciclovir Foscarnet	40	2/>1 log reduction	101	Yes	No	Alive
#30	M/31	R+	Bilateral lung	-	445	Yes(45,71312)/esophagitis & pneumonitis	Ganciclovir	6	2/>1 log reduction	61	Yes	Bacterial infection	Dead
#31	M/21	R+	Small intestine	ATG	416	No/enteritis	Ganciclovir	2	3/NA	NA	Yes	No	Dead
#32	F/53	R+	Liver		*0	Yes(67,191)/	Ganciclovir	1	7/unchanged	NA	Yes	IFI	Dead
#33	F/19	R+	Small intestine	Alemtuzumab	363	Yes(1,588)/colitis & enteritis	Ganciclovir Foscarnet	1	9/>1 log reduction	NA	No	IFI	Dead
#34	M/27	D+/R-	Small intestine	Alemtuzumab	256	Yes(1,259)/colitis & enteritis	Ganciclovir Foscarnet	3	39/>1 log reduction	151	No	No	Alive

- Recipients had CMV viremia at time of Transplant
- NA missing info

Table S2 HSCT recipients individual characteristics and outcome

Classification: Public - عام

Patient #	Sex/age	CMV serostatus	Primary disease/graft type	Conditioning regimen	Days transplant to CMV infection	CMV viremia(Peak IU/ML) /Disease	Days from CMV Diagnosis to CMVIG	Antiviral used	Number of doses/Response at day 14	Days to undetected CMV	GVHD/graft failure	Secondary infection	90 days mortality
#1	F/22	D+/R+	MPAL/allo	Myeloablative	275	Yes(47,702)	1	Ganciclovir	2/>1 log reduction	29	Liver GVHD in 1/2023 Eye GVHD 9/2024	Viral infection	Alive
#2	F/35	D+/R+	HL/allo	RIC Myeloablative	30	Yes(1,106)/pneumonitis	11	Ganciclovir	10/>1 log reduction	35	Gut GVHD 2/2021	IFI/bacterial infection	Dead
#8	M/25	D+/R+	ALL/Haplo	RIC Myeloablative	192	Yes(46,885)/colitis &pneumonitis	37	Ganciclovir foscarnet	4/>1 log reduction	23	None	IFI/Bacterial infection	Dead
#9	M/32	D+/R+	SCD/allo	Myeloablative	12	Yes(1,083)	4	Ganciclovir foscarnet	3/Unchanged	16	None	-	Alive
#11	M/67	D+/R+	AML/MUD	RIC Myeloablative	41	Yes(38,721)	5	Ganciclovir foscarnet	3/Undetected	13	Graft failure 5/2022	-	Lost follow up
#12	M/59	D+/R+	DLBCL/allo<br><br>CAR-T therapy prior to HSCT	Myeloablative	0	Yes(1,541)	1	Ganciclovir foscarnet	15/Unchanged	NA	None	IFI/viral infection	Dead
#13	F/51	D+/R+	ALL/Haplo	Myeloablative	27	Yes(6,830)	15	ValGanciclovir then letermovir 750 +CMVIG	1/Unchanged	18	Gut GVHD 9/2022 and Chronic graft failure	IFI/viral infection	Alive
#14	F/60	D+/R+	AML/Haplo	RIC Myeloablative	0	Yes(376)	1	Ganciclovir then ppx letermovir	2/Undetected	7	Relapsed AML post BMT 1/2023	-	Dead
#17	F/24	D+/R+	SCD/Haplo	Myeloablative	7	Yes(7,690)	10	Valganciclovir foscarent	5/>1 log reduction	56	None	Bacterial infection	Alive
#18	F/22	D+/R+	AA/Haplo	Myeloablative	315	Yes(1,564)	60	Foscarnet	3/Undetected	9	Skin GVHD3/2022	Bacterial infection	Alive
#20	M/31	D+/R+	SCD/allo	Myeloablative	20	Yes(387)	5	Letermovir 450	1/Unchanged	19	None	-	Alive
#22	M/29	D+/R-	SCD/allo	Myeloablative	43	Yes(9583)	2	Valganciclovir foscarent	3/>1 log reduction	17	None	-	Alive
#26	M/24	D-/R+ (Donor info missing)	AML/Haplo	Myeloablative	215	Yes(176,012)/encephalitis	9	Ganciclovir foscarnet	5/>1 log reduction	26	None	IFI	Lost follow up
#27	M/49	D+/R+	HL/allo	Myeloablative	11	Yes(4,231)	22	Letermovir then FOS then GCV Foscarnet	2/Unchanged	NA	Gut GVHD 6/2022	Bacterial infection	Dead
#28	F/56	D+/R+	CML/allo	Myeloablative	233	Yes(1,885)	22	Ganciclovir thenLetermovir	3/Unchanged	73	None	IFI/bacterial infection	Dead

AA Aplastic Anemia, MPAL Mixed phenotypic Acute Leukemia, ALL Acute lymphoid leukemia, AML Acute myeloid leukemia, SCD Sickle cell disease, HL Hodgkins lymphoma, DLBCL: Diffuse large B cell lymphoma, CML Chronic myeloid leukemia, FL Follicular lymphoma, MUD matched unrelated donor, Haplo Haploidentical HSCT, RIC Reduced intensity chemotherapy,

Table S3 CAR-T cell therapy patients characteristics and outcome

Patient #	Sex/age	CMV serostatus	Primary disease/graft type	Conditioning regimen	Days from CAR-T to CMV	CMV viremia(Peak IU/ML) /Disease	Days from CMV Diagnosis to CMVIG	Antiviral used	Number of doses/Response at day 14	Days to undetected CMV	Secondary infection	90 days mortality
#4	F/55	R+	DLBCL	Flu/Cy	1	Yes(16,974)/No	1	Foscarnet then Letermovir PPX when VL 353	1/>1 log reduction	115	IFI	Alive
#6	M/52	R+	HGBCL progressed to FL	Flu/CY	-7	Yes(52,274)/No	0	Ganciclovir Foscarnet then Letermovir PPX when VL 800	5/>1 log reduction	123	-	Dead
#23	M/42	R+	DLBCL	Flu/CY	-71	Yes(15,719)/No	23	Ganciclovir, ValGCV Foscarnet Ltermovir PPX when VL 246	5/>1 log reduction	52	IFI	Dead
#24	M/37	R+	DLBCL	Flu/CY	-3	Yes(3,065)/No	2	Ganciclovir Foscarnet	1/undetected	14	Bacterial infection	Dead

DLBCL: Diffuse large B cell lymphoma, HGBCL high grade B cell lymphoma, FL Follicular lymphoma, Flu/Cy fludarabine/ cyclophosphamide
